# Radiolucent Plastic Food Pick Ingestion in a Child: Endoscopic Retrieval Despite Negative CT Findings

**DOI:** 10.7759/cureus.89144

**Published:** 2025-07-31

**Authors:** Shun Imamura, Soichiro Ishimaru, Hikaru Maejima, Masayuki Hirai, Satoru Kisohara

**Affiliations:** 1 Department of Pediatrics, Kariya Toyota General Hospital, Kariya, JPN

**Keywords:** computed tomography, foreign body, pediatric endoscopy, plastic pick, radiolucent

## Abstract

Foreign body ingestion is a common pediatric emergency, and plastic objects are particularly difficult to detect on imaging due to their radiolucency. This can lead to delayed diagnosis and serious complications, especially when the ingestion is unwitnessed or the child is asymptomatic. We report a case of a four years and five months old girl who claimed to have accidentally swallowed a 4.6 cm tapered plastic food pick. She was asymptomatic on initial presentation, and neither X-rays nor CT scans revealed the foreign body. She was initially discharged. However, upon reevaluation of the history and characteristics of the object, a senior physician suspected the presence of a retained elongated object and advised re-presentation. Under general anesthesia, upper gastrointestinal endoscopy was performed, and the pick was safely retrieved from the stomach without complications. This case highlights the clinical significance of recognizing the potential danger posed by radiolucent, tapered plastic foreign bodies, even in asymptomatic patients. Clinical decisions should not rely solely on imaging but incorporate the history and nature of the object. This case emphasizes the importance of flexible clinical judgment and early intervention in managing radiolucent foreign body ingestion.

## Introduction

Foreign body ingestion is frequently encountered in pediatric emergency care, and the approach varies significantly depending on the nature and shape of the ingested object. Plastic materials, being radiolucent, are often not visible on X-rays or CT scans [1,2]. In cases with unclear witness accounts or mild symptoms, diagnosis and intervention may be delayed. Sharp or elongated foreign bodies can pose a high risk of complications, such as gastrointestinal perforation, even in the absence of symptoms. Therefore, clinical decisions should be based on a careful assessment of history and object characteristics, rather than solely on imaging findings. Here, we report a case of an asymptomatic child in whom a plastic foreign body was not visible on CT but was removed endoscopically based on clinical suspicion.

## Case presentation

**Figure 1:**
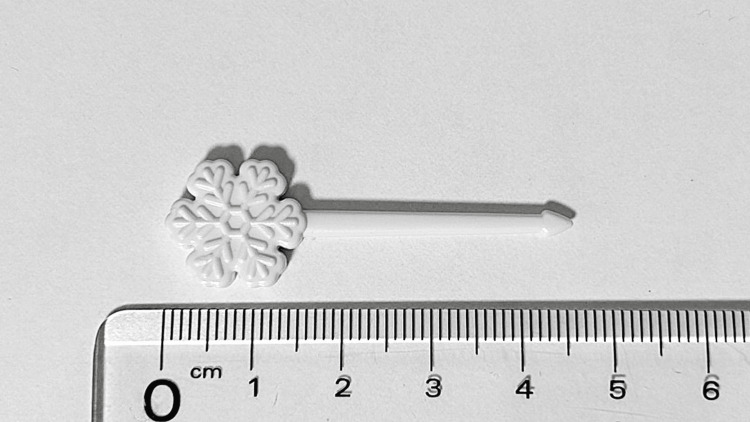
Ingested plastic pick The pick was made of plastic, with a maximum diameter of 1.6 cm at the decorative end and a total length of 4.6 cm.

A four years and five months old girl with no significant past medical or family history was brought to the emergency department approximately one hour after claiming she had accidentally swallowed a plastic food pick while playing with it. Although the event was unwitnessed by her parents, she complained of throat pain, and one pick was missing. On examination, she was asymptomatic, and physical findings of the pharynx and abdomen were unremarkable. Vital signs were within normal limits: body temperature 36.6 °C, heart rate 121 beats per minute, respiratory rate 15 breaths per minute, and oxygen saturation 99% on room air. The plastic pick brought by the family measured 4.6 cm and had a tapered tip (Figure 1).

**Figure 2:**
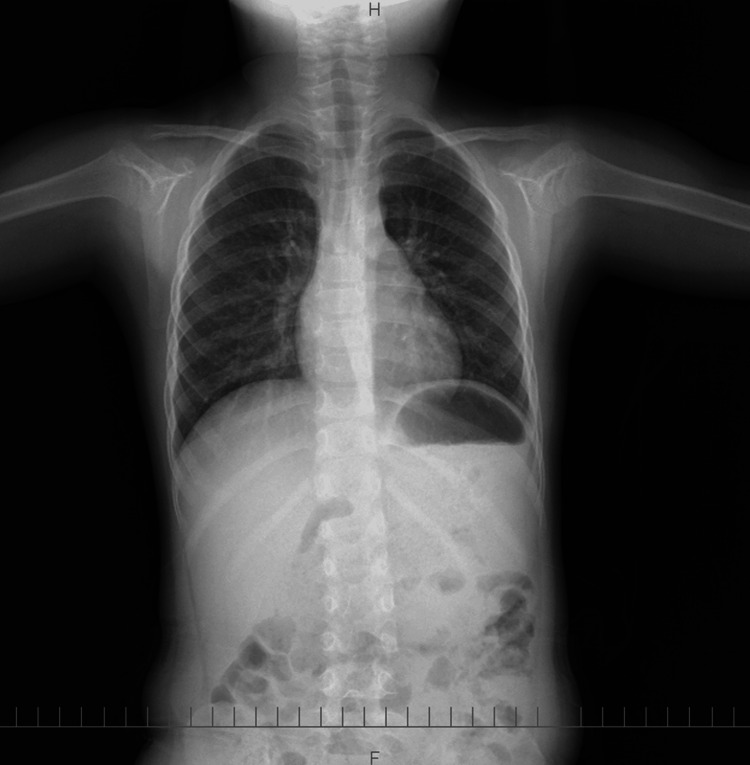
Chest X-ray No obvious foreign body could be identified.

**Figure 3:**
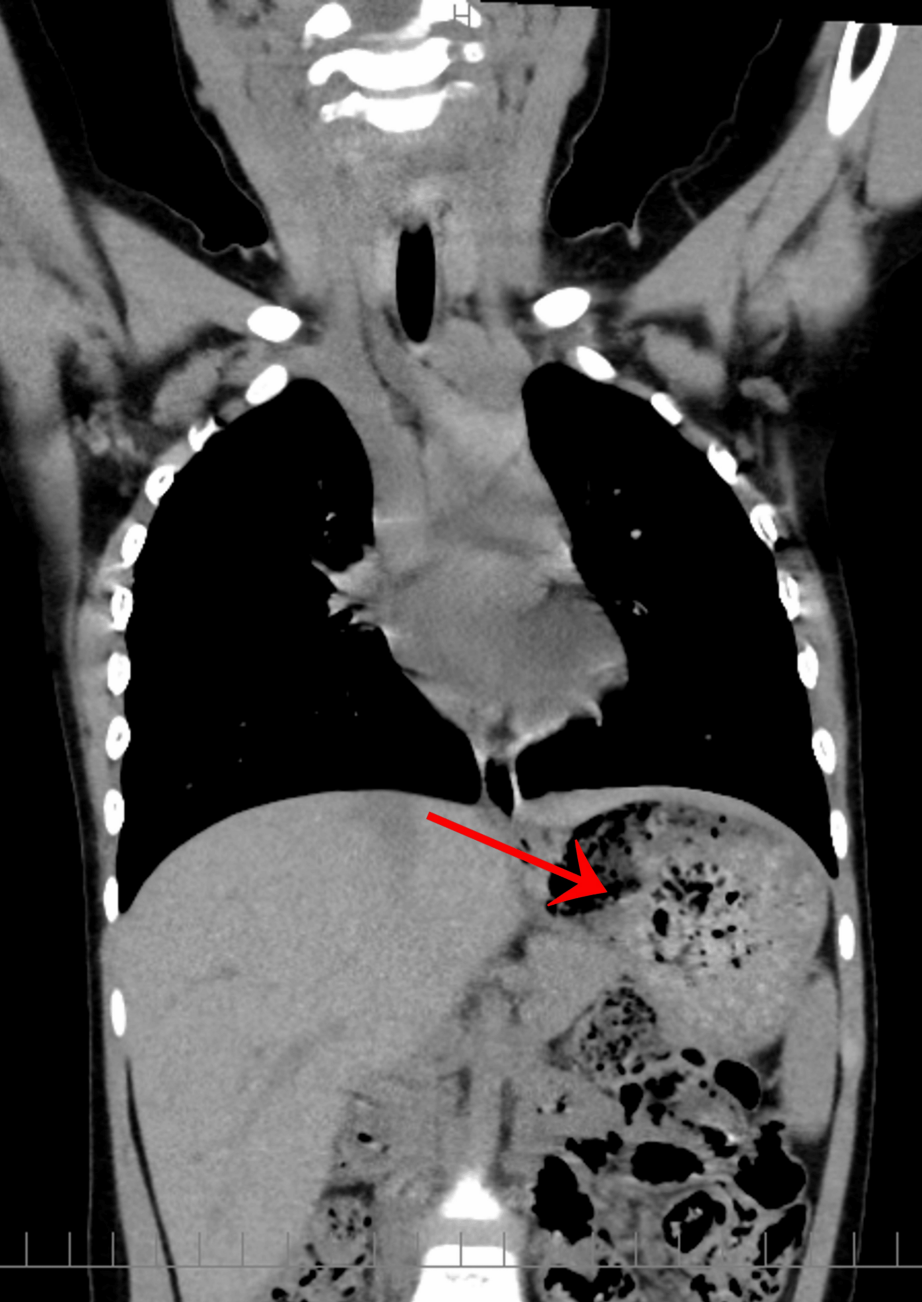
A CT scan from the neck to the abdomen There was a large amount of residual food in the stomach, but no foreign body could be identified.

Chest radiograph did not detect the object (Figure 2), and a non-contrast-enhanced CT scan from the neck to the abdomen also failed to reveal the foreign body or any signs of perforation (Figure 3). Given her asymptomatic status, the attending physician considered the likelihood of ingestion to be low and discharged the patient.

Subsequently, a senior physician re-evaluated the clinical history and object morphology, concluding that the pointed and elongated nature of the object made both retention and the risk of perforation highly probable. The caregiver’s account was deemed reliable. The family was contacted, and the child returned approximately four hours post-ingestion. Although still asymptomatic, there was concern that the object could have already passed into the small intestine. The potential for a negative endoscopy result despite general anesthesia was explained, but the caregivers opted for the procedure. Approximately two hours after her return to the hospital, upper gastrointestinal endoscopy under general anesthesia was performed in cooperation with the gastroenterology and anesthesiology departments.

**Figure 4:**
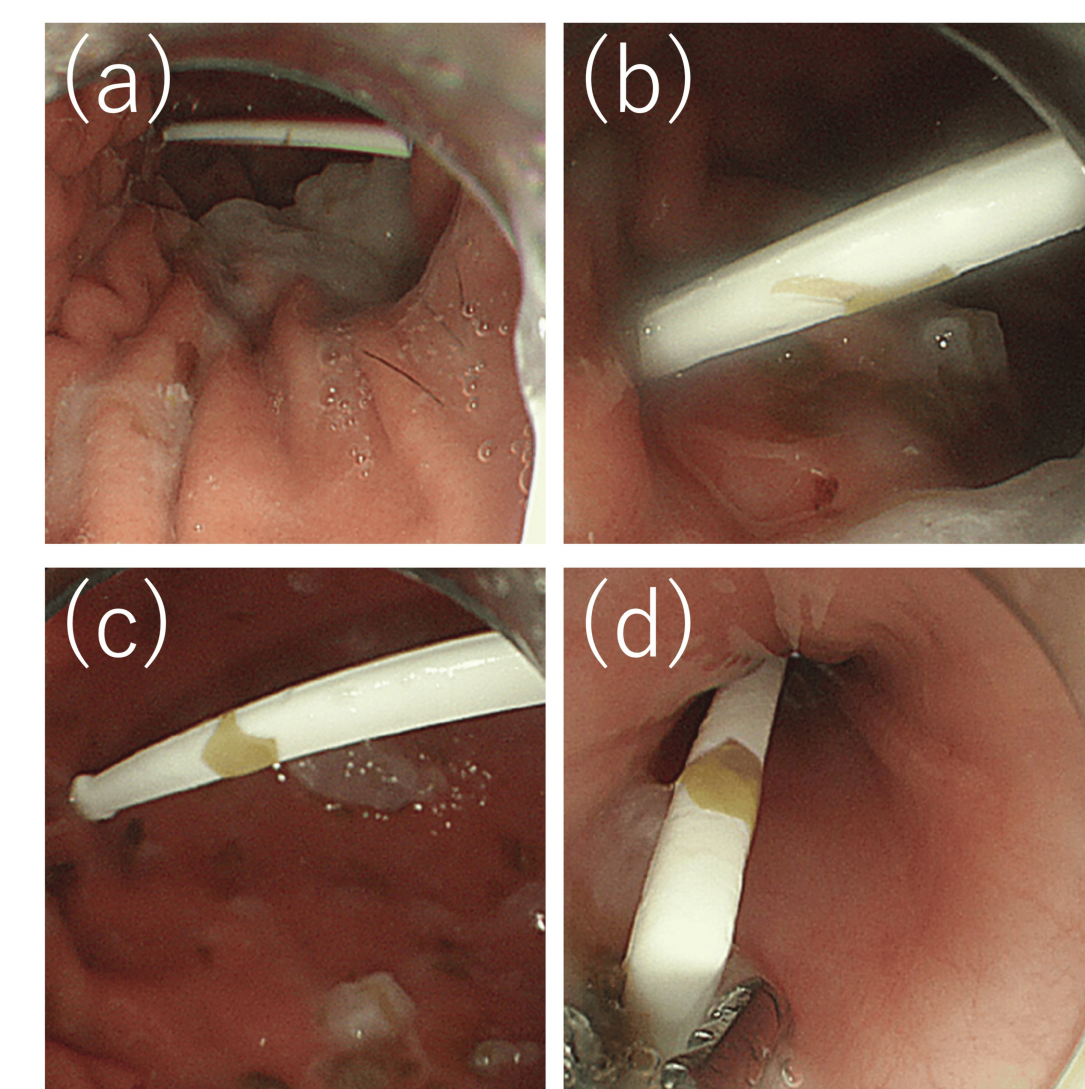
Endoscopic findings There was minimal residual food in the stomach, and only the plastic pick remained. Images (a)–(c) show the pick retained in the stomach, with its tapered tip pressing against the gastric wall. Image (d) shows the tip of the pick being grasped with alligator forceps in the esophagus during extraction.

Endoscopy revealed the plastic pick pressed against the gastric wall, with minimal residual food content in the stomach. The object was grasped and safely removed using alligator forceps (Figure 4). No bleeding or signs of perforation were observed. The postoperative course was uneventful, and the patient was discharged after resuming oral intake without symptoms.

## Discussion

This case involved a tapered, radiolucent plastic foreign body that was undetectable on imaging and removed following clinical judgment. Plastic foreign bodies are often radiolucent and may not be visible on X-rays or CT scans, which can lead to misdiagnosis or delays in diagnosis. Previous reports have documented gastrointestinal perforation despite normal imaging findings at the time of presentation [3-5]. Elongated, radiolucent objects may cause serious complications even in asymptomatic patients, underscoring the value of preventive intervention.

Management decisions for ingested foreign bodies depend on the material, shape, length, and sharpness. Guidelines from the European Society of Gastrointestinal Endoscopy (ESGE)/European Society for Paediatric Gastroenterology, Hepatology, and Nutrition (ESPGHAN) and the American Society for Gastrointestinal Endoscopy (ASGE) recommend endoscopic removal of foreign bodies longer than 5-6 cm, which are unlikely to pass spontaneously, even in asymptomatic patients [1,6,7]. However, since the reports that form the basis of the 5-6 cm threshold were conducted solely in adults, their applicability to infants or young children may be limited [8,9]. Hence, extrapolating these thresholds to young children may be inappropriate. Pediatric-specific criteria, such as the Colorado criteria, suggest that objects longer than 3 cm are anatomically unlikely to pass through the pylorus in infants, thereby supporting a lower threshold for removal in this population [10,11]. Furthermore, cases of perforation by plastic objects approximately 3-4 cm in length have also been reported [12]. In the present case, the foreign body measured 4.6 cm in a young child, and endoscopic retrieval was therefore considered appropriate.

With the increasing diversity of objects involved in pediatric foreign body ingestion, a Japanese cohort study by Fujisawa et al. found that 17% of ingested items were plastic [13]. Plastic food picks, commonly used in lunchboxes, are rarely reported as ingested items, but they pose significant risks. This case highlights that even everyday household items perceived as safe can pose serious hazards if ingested. Both caregivers and healthcare providers should be aware of this risk [13]. The CT detectability of plastic foreign bodies is variable and depends on multiple factors, including the type of polymer (e.g., high-density content), physical thickness, geometry, and CT imaging parameters such as window settings, orientation, and contrast resolution [14]. While thick and relatively dense plastics may be detectable on CT, thin, elongated, and low-density objects (such as in the present case) are often radiologically occult. Therefore, a negative CT scan should not be considered definitive evidence of the absence of a foreign body. Comprehensive clinical assessment, including ingestion history, circumstances, and object characteristics, remains essential. Although general anesthesia poses inherent risks, it was deemed appropriate in this case given the elongated nature of the object. Careful, case-by-case evaluation is essential when performing invasive procedures on asymptomatic pediatric patients. Accumulation of similar cases in the future may contribute to the establishment of clearer intervention criteria for radiolucent foreign bodies.

## Conclusions

This case highlights several important clinical considerations in the management of pediatric foreign body ingestion. Radiolucent objects such as plastic food picks may not be visible on imaging. Even in asymptomatic cases, early endoscopic intervention should be considered when ingestion is credible and the object is elongated.

However, the risks of invasive procedures, particularly under general anesthesia, must be carefully weighed. Accumulating similar cases will be important for defining and standardizing clinical criteria for intervention in radiolucent foreign body ingestion.
